# DNA methylation is associated with prenatal exposure to sulfur dioxide and childhood attention-deficit hyperactivity disorder symptoms

**DOI:** 10.1038/s41598-023-29843-y

**Published:** 2023-03-01

**Authors:** Yoon-Jung Choi, Jinwoo Cho, Yun-Chul Hong, Dong-wook Lee, Sungji Moon, Soo Jin Park, Kyung-shin Lee, Choong Ho Shin, Young Ah Lee, Bung-Nyun Kim, Zachary Kaminsky, Johanna Inhyang Kim, Youn-Hee Lim

**Affiliations:** 1 National Cancer Center Graduate School of Cancer Science and Policy, Goyang, Republic of Korea; 2 Department of Preventive Medicine, Seoul National University College of Medicine, Seoul, Republic of Korea; 3 Environmental Health Center, Seoul National University College of Medicine, Seoul, Republic of Korea; 4 Department of Statistics, University of Pittsburgh, Pittsburgh, PA USA; 5 Institute of Environmental Medicine, Seoul National University Medical Research Center, Seoul, Republic of Korea; 6 Public Healthcare Center, Seoul National University Hospital, Seoul, Republic of Korea; 7 Department of Surgery, Wonkwang University Sanbon Hospital, Gunpo, Republic of Korea; 8 Public Health Research Institute, National Medical Center, Seoul, Republic of Korea; 9 Department of Pediatrics, Seoul National University College of Medicine, Seoul National University Children’s Hospital, Seoul, Republic of Korea; 10 Division of Children and Adolescent Psychiatry, Department of Psychiatry, Seoul National University Hospital, Seoul, Republic of Korea; 11 Institute of Mental Health Research, University of Ottawa, Ottawa, Canada; 12 Department of Cellular and Molecular Medicine, University of Ottawa, Ottawa, Canada; 13 Department of Psychiatry and Behavioral Sciences, Johns Hopkins University School of Medicine, Baltimore, USA; 14 Department of Psychiatry, Hanyang University Medical Center, 222-1 Wangsimni-Ro, Seongdong-Gu, Seoul, 04763 Republic of Korea; 15 Section of Environmental Epidemiology, Department of Public Health, University of Copenhagen, Østerster Farimagsgade 5, 1014 København K, Copenhagen, Denmark

**Keywords:** Epidemiology, Molecular medicine

## Abstract

Epigenetic influence plays a role in the association between exposure to air pollution and attention deficit hyperactivity disorder (ADHD); however, research regarding sulfur dioxide (SO_2_) is scarce. Herein, we investigate the associations between prenatal SO_2_ exposure and ADHD rating scale (ARS) at ages 4, 6 and 8 years repeatedly in a mother–child cohort (n = 329). Whole blood samples were obtained at ages 2 and 6 years, and genome-wide DNA methylation (DNAm) was analyzed for 51 children using the Illumina Infinium HumanMethylation BeadChip. We analyzed the associations between prenatal SO_2_ exposure and DNAm levels at ages 2 and 6, and further investigated the association between the DNAm and ARS at ages 4, 6 and 8. Prenatal SO_2_ exposure was associated with ADHD symptoms. From candidate gene analysis, DNAm levels at the 6 CpGs at age 2 were associated with prenatal SO_2_ exposure levels. Of the 6 CpGs, cg07583420 (*INS-IGF2*) was persistently linked with ARS at ages 4, 6 and 8. Epigenome-wide analysis showed that DNAm at 6733 CpG sites were associated with prenatal SO_2_ exposure, of which 58 CpGs involved in Notch signalling pathway were further associated with ARS at age 4, 6 and 8 years, persistently. DNAm at age 6 was not associated with prenatal SO_2_ exposure. Changes in DNAm levels associated with prenatal SO_2_ exposure during early childhood are associated with increases in ARS in later childhood.

## Introduction

Attention deficit hyperactivity disorder (ADHD) is a neurobehavioral disorder characterized by inattentiveness, hyperactivity, and impulsivity^1^. Children with ADHD have difficulties in learning, family relationships, and social interaction^2^. ADHD affects 5–7% of children and adolescents worldwide^3,4^. Furthermore, at least 5% of children do not meet the full diagnostic criteria despite exhibiting ADHD symptoms^5^. ADHD in children often persists into late adolescence and adulthood, which is a risk factor of other mental health issues, including antisocial behaviors, self-harm, and substance misuse^5^.

Although the etiology of ADHD is largely unknown and complex, it is characterized by numerous gene-environmental interactions^6^. Preconceptional, gestational, and perinatal conditions have been indicated to affect ADHD^6^. Maternal nutrition during pregnancy, pesticides and heavy metal exposure during gestation, premature birth have been reported as risk factors of ADHD in children^6^. Prenatal exposure to air pollution is also a potential risk factor for ADHD^7,8^. Previous studies have reported an association between children’s behavioral development and prenatal exposure to sulfur dioxide (SO_2_)^9,10^, one of the major gaseous pollutants derived from coal-fired power plants, smelters, and industrial emissions^11^. Although the relation between SO_2_ exposure and ADHD has not been well investigated, SO_2_ has been associated with various neurodevelopmental deficits, such as fine motor skills, executive function, and ADHD-related hospital admissions^12,13^.

Epigenetic processes such as DNA methylation (DNAm) have been proposed to underly the association between environmental exposures and ADHD^14^. A previous study has demonstrated that methylation differences of the growth factor-independent 1 transcriptional repressor (*GFI1*) region partially mediated the association between maternal smoking during pregnancy and ADHD symptoms at age 6^15^. Another study found that long-term prenatal exposure to paracetamol (acetaminophen) is associated with DNAm differences in children diagnosed with ADHD^16^. Recent evidence suggests that air pollution may affect methylation through an oxidative stress pathway^17,18^. Air pollution-induced reactive oxygen species oxidize 5-hydroxymethylcytosine causing DNA demethylation^19^. It also leads to the hypomethylation of CpG cytosine residues by inhibiting the activity of methyltransferases via alteration in their sequence alignment to the corresponding base sequences of the DNA^20^. To date, no study has investigated the mediation effect of DNAm on the association between SO_2_ and ADHD.

In this study, we aimed to (1) examine the association between prenatal exposure to SO_2_ and ADHD symptoms at multiple ages during childhood and (2) investigate the effect of DNAm on the association between SO_2_ exposure and ADHD symptoms. We first analyzed the association between prenatal SO_2_ and ADHD symptoms at ages 4, 6 and 8, which are critical periods of symptom manifestation. Then, we identified CpG sites (CpGs) associated with both prenatal SO_2_ exposure and ADHD symptoms during childhood. We utilized both candidate gene analysis approach by targeting CpGs associated with ADHD symptoms, and an epigenome-wide association study. We also aimed to detect co-methylated CpGs from a module correlated with both prenatal SO_2_ exposure and ADHD symptoms using weighted gene co-metylation network analysis (WGCNA).

## Results

### General characteristics of study participants

**Figure 1:**
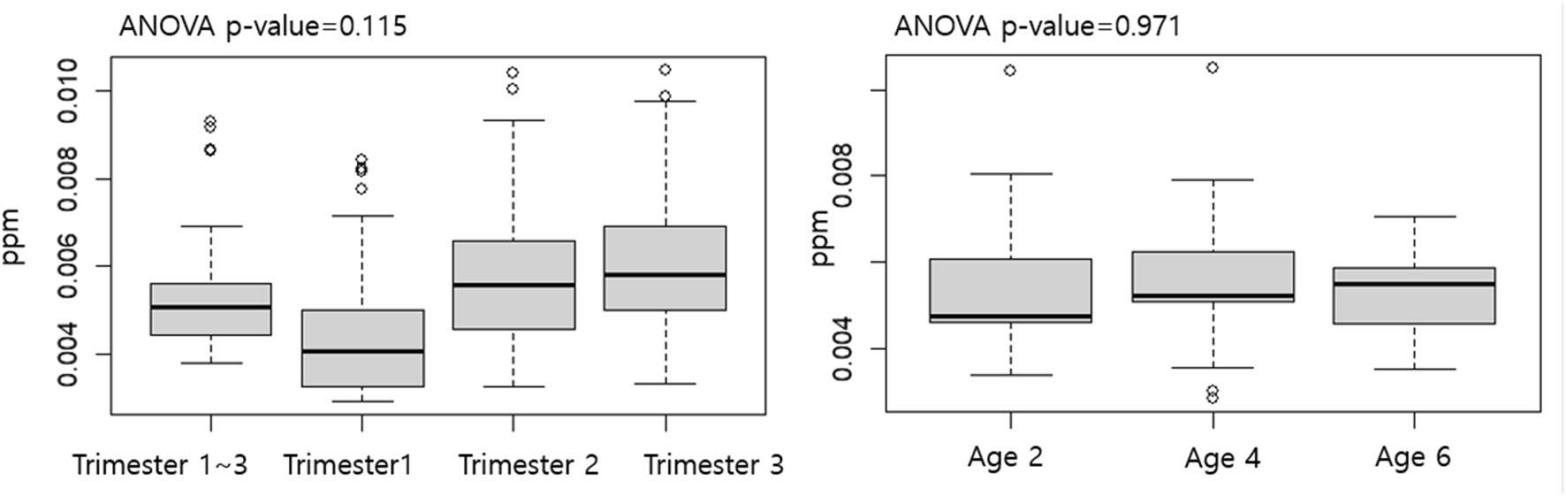
The prenatal SO_2_ exposure according to the trimester (left) and postnatal SO_2_ exposure according to the ages of 2, 4 and 6 years (right).

There were significant differences in maternal smoking and SO_2_ exposure during the 1^st^ trimester between the entire cohort and the sub-cohort (Table 1). Other covariates were not significantly different between the entire cohort and sub-cohort. The mean age of mothers at pregnancy was 31.3 years, most of the mothers were college graduates (70.5–71.7%), while 76.9–79.6% of mothers were exposed to environmental tobacco smoking (ETS) during pregnancy. Mean maternal IQ was 116–118. Mean maternal SO_2_ exposure during pregnancy was 0.0044–0.0060 ppm depending on the trimester. Among children, 51.0–53.5% were girls, 86.3–90.3% were singleton, 88.0–91.8% were born full-term. Mean postnatal SO_2_ exposure ranged from 0.052 to 0.058 ppm depending on the ages. Mean ADHD rating scale (ARS) was 5.96–6.96 at age 4, 5.87–6.04 at age 6, and 5.57–6.34 at age 8. In the sub-cohort, prenatal and postnatal SO_2_ exposure were not significantly different according to the trimester or ages (Fig. 1).

**Table 1:** General characteristics of study participants.

Participants	Variables	Category	Total cohort (n = 329)	Sub-cohort (n = 51)	p-value
Mother	Age at pregnancy		31.5 ± 3.51	31.3 ± 3.53	0.670
	Educational level	Middle school graduate	1 (0.3%)	0	< 0.001
		High school graduate	55 (16.7%)	7 (13.7%)	
		College graduate	236 (71.7%)	36 (70.5%)	
		Graduate school	37 (11.2%)	8 (15.75)	
	Smoking status during pregnancy	Never smoked	250 (76.0%)	27 (54.0%)	< 0.001
		Smoked before pregnancy	61 (18.5%)	18 (36.0%)	
		Smoked during pregnancy	6 (1.82%)	5 (10.0%)	
	Environmental tobacco smoking	Never exposed	62 (18.8%)	10 (20.5%)	0.906
		Exposed	253 (76.9%)	39 (79.6%)	
	IQ		116 ± 11.1	118 ± 11.2	0.257
	SO_2_ exposure (ppm)	1st–3rd trimester	0.0054 ± 0.0015	0.0053 ± 0.0012	0.516
		1st trimester	0.0052 ± 0.0016	0.0044 ± 0.0014	< 0.001
		2nd trimester	0.0056 ± 0.0019	0.0058 ± 0.0015	0.408
		3rd trimester	0.0056 ± 0.0020	0.0060 ± 0.0014	0.088
Child	Sex	Male	176 (53.5%)	25 (49.0%)	0.551
		Female	153 (46.5%)	26 (51.0%)	
	Season of birth	Spring	69 (21.0%)	9 (17.6%)	< 0.001
		Summer	115 (35.0%)	31 (60.8%)	
		Autumn	78 (23.7%)	11 (21.6%)	
		Winter	67 (20.4%)	0	
	Multiple birth	Singleton	297 (90.3%)	44 (86.3%)	0.381
		Multiple birth	32 (9.73%)	7 (13.7%)	
	Preterm (< 37 weeks)	No	301 (91.8%)	44 (88.0%)	0.379
		Yes	27 (8.23%)	6 (12.0%)	
	SO_2_ exposure (ppm)	2 years	0.0057 ± 0.0014	0.0053 ± 0.0013	0.031
		4 years	0.0058 ± 0.0013	0.0056 ± 0.0013	0.292
		6 years	0.0052 ± 0.0011	0.0053 ± 0.00089	0.507
	ADHD rating scale	4 years	6.96 ± 5.95	5.96 ± 4.61	0.171
		6 years	5.87 ± 5.47	6.04 ± 6.55	0.863
		8 years	6.34 ± 5.53	5.57 ± 5.43	0.351
	Cell type fraction	CD4 T cell	NA	0.158 ± 0.0430	NA
		CD8 T cell	NA	0.203 ± 0.0445	NA
		NK cell	NA	0.0415 ± 0.0386	NA
		B cell	NA	0.184 ± 0.0470	NA
		Monocyte	NA	0.0462 ± 0.0257	NA
		Neutrophil	NA	0.359 ± 0.0858	NA

### Selection of candidate CpGs

After the systematic review, a total of 22 studies were selected^17,21–41^ (Table S1). A total of 597 CpGs were pooled from these studies (Table S2), and final 375 CpGs were selected for analysis. The detailed result of the systematic review and selection of candidate CpGs is presented in Supplementary Materials and Fig. S1.

### Association between prenatal exposure to SO_2_ and ADHD symptoms

In the entire cohort (n = 329), one interquartile range (IQR) increase in prenatal SO_2_ exposure during the 1st trimester of pregnancy was significantly associated with 8.38% (95% CI 3.19, 13.83) increase in ARS at age 4; however, such association was not observed at age 6 or 8 (Table 2). The associations between prenatal SO_2_ exposure during the entire period or 2nd trimester of pregnancy and childhood ADHD symptoms at ages 4, 6 and 8 were not significant. Prenatal SO_2_ exposure during the 3rd trimester of pregnancy was not associated with ADHD symptoms at ages 4 or 6, but was negatively associated with ADHD symptoms at age 8.

**Table 2:** Prenatal SO_2_ exposure and ADHD rating scale at 4, 6 and 8 years of age in the cohort (n = 329).

Trimester	% Change (95% CI)	p-value	% Change (95% CI)	p-value	% Change (95% CI)	p-value
	4 years		6 years		8 years	
1st–3rd	2.68 (− 0.62, 6.09)	0.113	− 1.14 (− 4.85, 2.71)	0.557	0.23 (− 3.28, 3.86)	0.901
1st	8.38 (3.19, 13.83)	0.001	3.97 (− 1.69, 9.95)	0.173	5.09 (− 0.41, 10.91)	0.07
2nd	3.68 (− 1.67, 9.32)	0.181	− 3.66 (− 9.33, 2.35)	0.227	4.04 (− 1.59, 10)	0.163
3rd	− 1.91 (− 7.14, 3.63)	0.492	− 4.68 (− 10.45, 1.46)	0.132	− 7.39 (− 12.73, − 1.73)	0.011

Adjusted for maternal age at pregnancy, mother’s education levels, maternal smoking, maternal environmental tobacco smoking, season of child’s birth.

### Association between prenatal exposure to air pollution and DNAm

**Figure 2:**
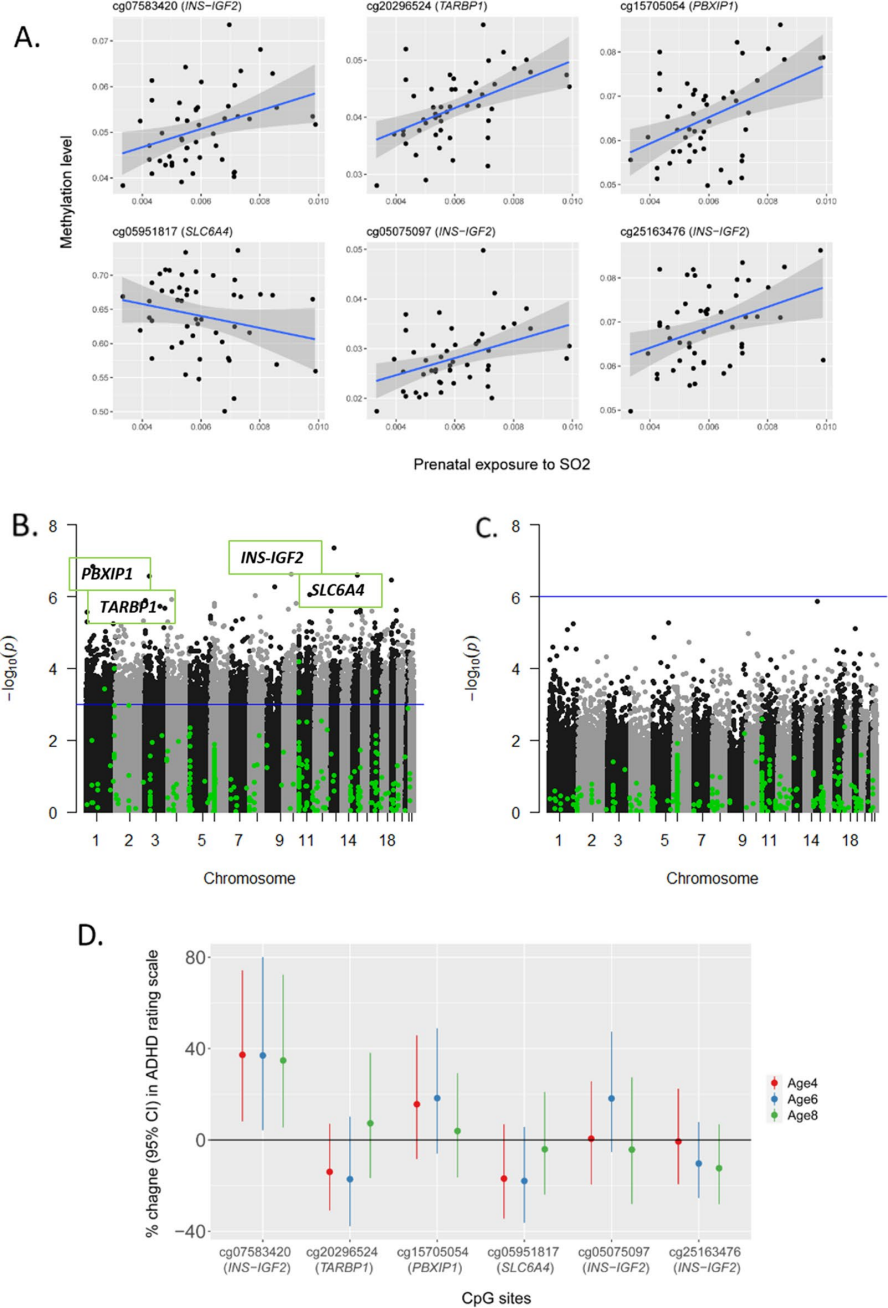
The association between prenatal SO_2_ exposure, DNA methylation, and ADHD rating score. (**A**) The association between prenatal SO_2_ exopusre and DNA methyhlation. (**B**) Manhattan plot showing the association between prenatal SO_2_ exposure during the 3rd trimester and DNA methylation at age 2. The green dots indicate CpGs selected from literature review for a candidate gene analysis. The blue line is the threshold for FDR-corrected p-value < 0.05. The CpGs significantly associated with prenatal SO_2_ exposure from the candidate gene analysis were above the threshold line (cg07583420 (*INS-IGF2*), cg20296524 (*TARBP1*), cg15705054 (*PBXIP1*), cg05075097 (*INS-IGF2*), and cg25163476 (*INS-IGF2*)). (**C**) Manhattan plot showing the association between prenatal SO_2_ exposure during the 3rd trimester and DNA methylation at age 6. (**D**) The association between DNA methylation at the CpGs linked with prenatal SO_2_ exposure and the ADHD rating scale at ages 4, 6 and 8.

DNAm at age 2 at the candidate CpGs was not associated with prenatal SO_2_ exposure at the 1st–3rd, 1st, or 2nd trimester of pregnancy (Tables S3–S5). However, DNAm in children at age 2 at cg07583420 (*INS-IGF2*), cg20296524 (*TARBP1*), cg15705054 (*PBXIP1*), cg05075097 (*INS-IGF2*), and cg25163476 (*INS-IGF2*) were positively associated with an IQR increase in maternal SO_2_ exposure during the 3^rd^ trimester of pregnancy (Table 3, Fig. 2A, Table S6). DNAm at cg05951817 (*SLC6A4*) was negatively associated with SO_2_ exposure during the 3^rd^ trimester. DNAm at the 375 candidate CpGs in children at age 6 was not associated with prenatal SO_2_ exposure in any trimester (Tables S7–S10). We also conducted the epigenome-wide analysis for 326,898 CpGs to investigate the association between prenatal SO_2_ exposure and DNAm at age 2. We found that a total of 6,733 CpG sites were associated with prenatal exposure during the 3rd trimester with FDR-corrected p-value < 0.05 (Table S11, Fig. 2B), and the 6 CpGs listed above were included in the 6733 CpGs. Conversely, neither candidate gene analysis nor EWAS yielded significant CpGs within FDR-corrected p-value < 0.05 at age 6 (Fig. 2C).

**Table 3:** Association between prenatal exposure to sulfur dioxide during the 3rd trimester (per IQR increase) and DNAm at ADHD associated CpGs at 2 years of age* (FDR corrected p-value < 0.05) (n = 51).

CpGs	Chr	Gene	Gene group	Relation to CpG island	Estimate	p-value (BH corrected p-value)
cg07583420	11	*INS-IGF2*	Body	N shore	0.00683	6.48☓10^−5^(0.025)
cg20296524	1	*TARBP1*	1st exon	Island	0.00588	1.03☓10^−4^ (0.025)
cg15705054	1	*PBXIP1*	5’ UTR		0.00812	3.66☓10^−4^ (0.039)
cg05951817	17	*SLC6A4*	5’ UTR	N shore	− 0.0385	4.54 ☓10^−4^ (0.039)
cg05075097	11	*INS-IGF2*	Body	N shelf	0.00548	4.62 ☓10^−4^ (0.039)
cg25163476	11	*INS-IGF2*	Body	Island	0.00777	4.81 ☓10^−4^ (0.039)

*IQR*, interquartile range, *FDR* false discovery rate, *ADHD* attention-deficit hyperactivity disorder.

*Adjusted for mother’s age, education level, maternal smoking, maternal environmental tobacco smoking, and season of birth.

### Association between postnatal SO_2_ exposure and DNAm

The associations between the postnatal SO_2_ exposures at age of 2 years and DNAm at age 2, or the association between the postnatal SO_2_ exposure at ages 2, 4 or 6 years and DNAm at age 6 were not significant (Tables S12–S15). The associations between the cumulative postnatal SO_2_ exposures from age 2 to 4 or from 2 to 6 and DNAm at age 6 were not significant (Tables S16,S17).

### Association between DNAm and ADHD symptoms

Among the 6 CpGs with significant associations at age 2 with prenatal SO_2_ exposure, DNAm level at cg07583420 (*INS-IGF2*) were associated with 37.25% (95% CI 8.12, 74.22), 37.00% (95% CI 4.23, 80.08), and 34.82% (95% CI 5.44, 72.40) increase in ARS at ages 4, 6 and 8, respectively (Fig. 2D, Table S18).

Among 6733 CpGs from EWAS between prenatal SO_2_ exposure during the 3rd trimester and DNAm, 58 CpGs, 219 CpGs, and 2063 CpGs were associated with ARS at ages 4, 6, and 8 years, respectively (Tables S19–S21). Among these, DNAm at 58 CpGs was persistently associated with ARS at ages 4, 6 and 8 years (Fig. S2).

### Mediation analysis

**Figure 3:**
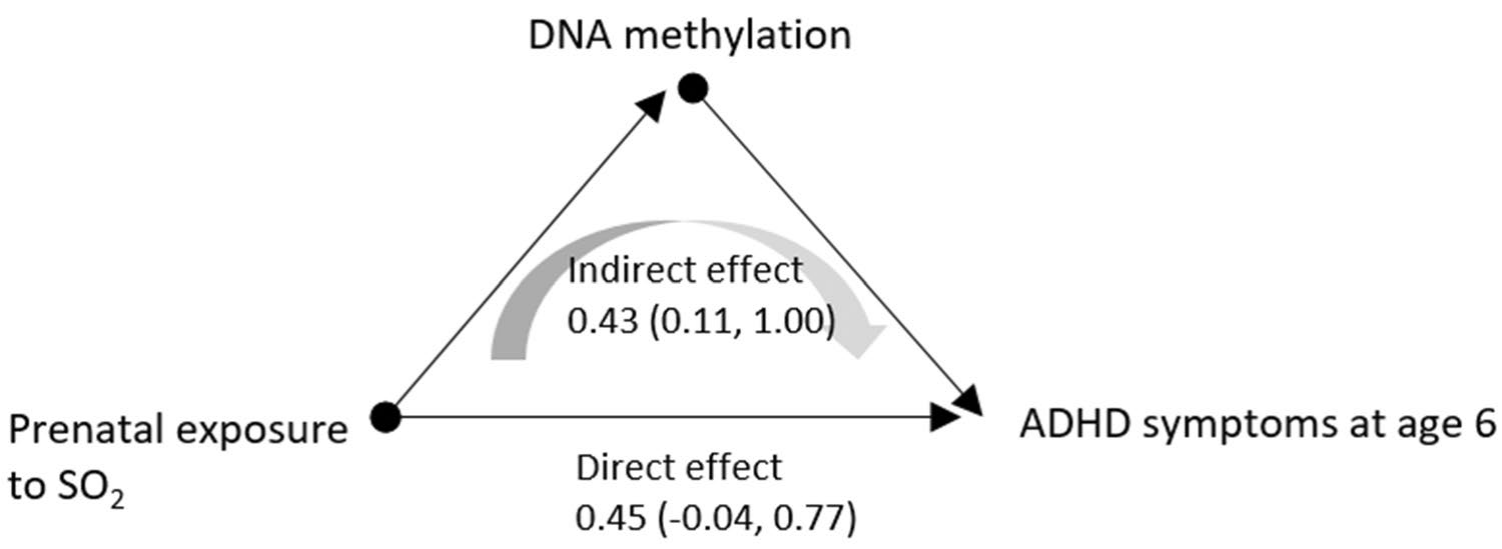
Mediation analysis showing the association between prenatal exposure to SO_2_ and ADHD symptoms in children through DNA methylation at cg07583420 (*INS-IGF2*).

The indirect effect of prenatal exposure during the 3^rd^ trimester on ARS in childhood through DNAm at cg07583420 (*INS-IGF2*) was positive and significant at ages 4 (p-value 0.028) and 6 (p-value 0.004) but not significant at age 8 (Table 4, Fig. 3). Direct effects, from prenatal exposure to childhood ARS, were marginally significant at ages 6 (p-value 0.060) and 8 (p-value < 0.001). Particularly at age 6, indirect effect, direct effect, and total effect (indirect effect + direct effect) were all positive and significant or marginally significant.

**Table 4:** Mediation analysis of the path from prenatal exposure to SO_2_ to ADHD rating scale (ARS) at 4, 6 and 8 years of age through DNAm at cg07583420 (*INS-IGF2*).

	4 years		6 years		8 years	
	Estimate (95% CI)	p-value	Estimate (95% CI)	p-value	Estimate (95% CI)	p-value
Indirect effect*	1.02 (0.11, 2.61)	0.028	0.43 (0.11. 1.00)	0.004	− 0.01 (− 0.17, 0.14)	0.890
Direct effect^†^	− 1.60 (− 6.14, 0.28)	0.144	0.45 (− 0.04, 0.77)	0.060	0.65 (0.45, 0.92)	< 0.001
Total effect^‡^	− 0.58 (− 3.53, 0.73)	0.748	0.88 (0.65, 1.12)	< 0.001	− 0.01 (− 0.24, 0.20)	0.890

*Indirect effect refers to prenatal exposure affecting ARS through DNAm at cg0783420.

^†^Direct effect refers to prenatal exposure directly affecting ARS not through DNAm.

^‡^Total effect refers to the sum of indirect effect and direct effect.

### Identifying methylation quantitative trait loci (mQTL)

A total of 60 SNPs were found within 100 kb from either side from cg07583420 (*INS-IGF2*). Four SNPs showed significant association with the DNA methylation levels at cg07583420 (p-value < 0.05): rs139951739, rs6578246, rs17885785, and rs3213223. However, none was significant when corrected for multiple comparisons using FDR method (Table S22, Fig. S3).

### Pathway enrichment analysis

Reactome pathways analysis showed that mostly NOTCH signalling was involved in genes annotated to the 58 CpGs associated with prenatal SO_2_ exposure during the 3rd trimester and ARS through 4, 6 and 8 years (Table S23, Figure S4).

### WGCNA

**Figure 4:**
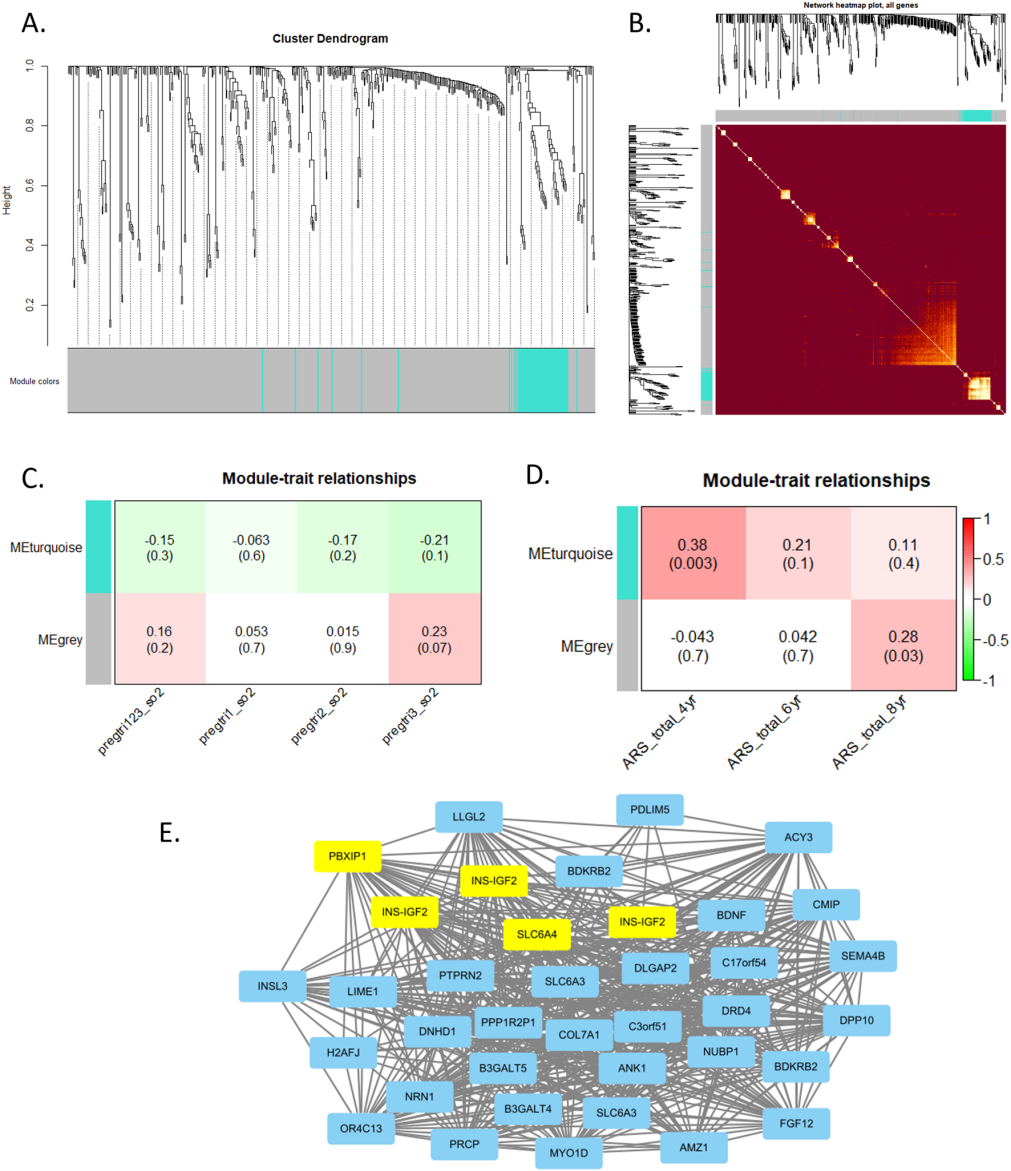
Weighted gene co-methylation network analysis of DNA methylation at CpGs for candidate gene analysis. (**A**) Dendrogram produced by hierarchical chlustering of samples. Modules of co-methylated CpGs are indicated by different color (grey and turquoise) beneath the dendrogram. (**B**) Network heatmap plot. Dendrograms from hierarchical clustering are corresponded to the color-coded modules (grey and turquoise). In the heatmap, bright yellow color indicate high co-expression inter-connectedness. (**C**) Correlating modules with exposure variables (prenatal SO2 exposure by trimester). (**D**) Correlating modules with outcome variable (ADHD rating scales at age 4, 6 and 8). (**E**) Network between CpGs from the turquoise module.

A total of 375 CpGs were analyzed for co-methylation. Two modules were detected: grey (316 CpGs) and turquoise (59 CpGs) (Fig. 4A,B). The correlation between the grey module and prenatal exposure to SO_2_ during the 3rd trimester was not significant (Fig. 4C). CpGs from the grey modules were analyzed again for co-methylation, and the turquoise module was correlated with ARS at age 4 (Pearson’s coefficient correlation 0.38, p-value 0.003), and the grey module was correlated with ARS at age 8 (Pearson’s coefficient correlation 0.28, p-value 0.03) (Fig. 4D). CpGs from the final turquoise module were assessed, and the CpGs associated with SO_2_ exposure during the 3rd trimester of pregnancy including *INS-IGF2*, *PBXIP1*, and *SLC6A4* (Table 3) were found from the turquoise module (Fig. 4E), suggesting that these CpGs are co-methylated in relation with ARS at age 4.

## Discussion

**Figure 5:**
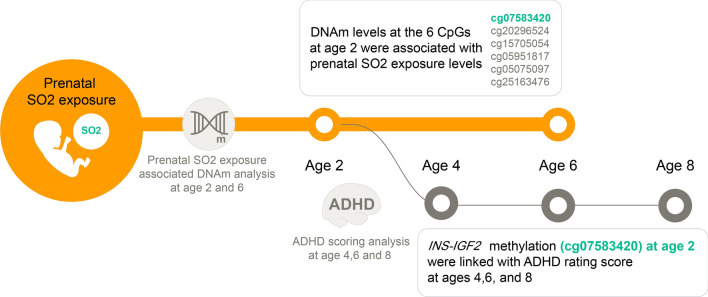
The overview of the study design and results (Candidate gene analysis).

To the best of our knowledge, this is the first study to report the epigenetic effect on the positive association between prenatal SO_2_ exposure and ADHD symptoms at ages 4, 6 and 8 years in a mother–child cohort. For a candidate gene analysis, we selected 375 CpGs from a systematic literature review of EWAS of ADHD. In the sub-cohort with available DNAm measurements at age 2, an IQR increase in prenatal SO_2_ exposure during the 3rd trimester was associated with an increased level of DNAm at CpGs of *INS-IGF2*, *TARBP1*, *PBXIP1*, and *SLC6A4*. Among these, DNAm level at cg07583420 (*INS-IGF2*) was positively associated with ARS at ages 4, 6 and 8, persistently (Fig. 5). When significant CpGs (n = 6733) from EWAS were tested for the association with ARS, 58 CpGs were persistently associated with ARS at ages 4, 6 and 8 years, which were involved in NOTCH signalling pathway. DNAm at age 6 was not associated with prenatal SO_2_ exposure. We further investigated the association between postnatal exposure to SO_2_ and DNAm by several different approaches, which showed no significant result. This implies that prenatal SO_2_ exposure may affect DNAm more significantly than postnatal SO_2_ exposure.

Prenatal environmental exposure may affect ADHD development through DNAm, which may lead to long-term phenotype changes^42^. The fact that DNAm was associated with prenatal SO_2_ at age 2 but not at age 6 may be related to the trajectory of global DNAm over the developmental stages, which fluctuates at an earlier stage of development and later stabilized^43^. This implies that a sensitive window period of DNAm may exist in which DNAm at earlier childhood is more susceptible to environmental triggers than later childhood^44^. For example, DNAm at several CpGs at age 7 was mostly predicted by early childhood adversity before age 3^45^. To investigate the period of clinical manifestation of ADHD with respect to DNAm, we examined ADHD symptoms at ages 4, 6 and 8. Surprisingly, DNAm was associated with ADHD symptoms during the wide range of early childhood from ages 4 to 8 consistently rather than focal stage of childhood, suggesting that the effect of DNAm can extend to a long period.

DNAm is also under genetic control if mQTL is identified adjacent to cg07583420 (*INS-IGF2*). In the present study, we could not identify statistically significant mQTL, possibly due to the small sample size. Further studies with a larger sample size are therefore warranted to elucidate the complex interplay between genetic, epigenetic, and environmental factors in the etiology of ADHD in children.

Although ADHD is associated with various compounds of air pollution, studies on the relationship between SO_2_ and neurodevelopmental outcomes, like ADHD, are limited. SO_2_ exposure prenatally up to 12 months was negatively associated with fine motor scores at 18 months of age^12^, and SO_2_ exposure was related to poor executive function in 6–12-year-old children in a cross-sectional study in China^12^. Another study reported an association between short-term exposure to SO_2_ and ADHD-related hospital admission in adolescents aged 10–19 years^13^.

The biological mechanism underlying the association between SO_2_ exposure and ADHD remains unknown. Therefore, only a few hypothetical mechanisms can be inferred from previous studies. SO_2_ can increase lipid peroxidation in the brain and generate reactive oxygen species^46^, as well as various inflammatory cytokines. These reactive oxygen species and inflammatory cytokines can relocate to the central nervous system via systemic circulation, thereby inducing neuroinflammation. SO_2_ has also been reported to induce neurotoxicity through protein oxidation, DNA protein cross-links, apoptosis, and damage of cell constituting the central nervous systems including cerebral cortex neurons, glial cell and nerve fibers^47,48^.

Another possible link is the association between SO_2_ and other regional pollutants. The region close to power plants or industrial facilities using fossil fuel shows higher SO_2_ level^49^. A cohort study with 5193 children including 116 patients with ADHD reported that the proximity to an industrial estate in the study area was associated with an increased risk of ADHD, and suggested prenatal exposure to organochlorine compound released from the industrial estate as a potential explanation for the finding^50^.

Notably, the result of WGCNA showed that the CpGs (cg07583420 (*INS-IGF2*), cg15705054 (*PBXIP1*), cg05075097 (*INS-IGF2*), and cg25163476 (*INS-IGF2*)) associated with prenatal SO_2_ exposure were co-methylated, corresponding to the module simultaneously correlated with prenatal SO_2_ during the 3rd trimester and ARS at age 4. *IGF2* plays an integral role in brain development after birth^51^. *IGF2* is associated with developmental abnormalities in the structure and/or function of the cerebellum and the hippocampus^52,53^, both of which are associated with ADHD. Higher *IGF2* methylation can predict ADHD symptoms in youth with conduct disorders^33^. In mice, *IGF2* enhancer deletion disrupted levels of striatal dopamine, which has been suggested to be involved in the pathophysiology of ADHD^54^.

Notch signalling pathway plays an essential role in embryogenesis and organogenesis^55^, particularly in regulation of neurogenesis^56^, and is also known to be involved in schizophrenia and bipolar disorder^57^. In a mice model, damages in neural stem cells affected cognitive impairment during Pb exposure, which was dependent on the Notch pathway^58^. Prenatal exposure to ketamine in rats, which increased the expression level of Notch1, inhibited the proliferation and differentiation of neural stem cells in hippocampus and impaired neurocognitive function including learning and memory in adulthood^56^.

Our study presents certain limitations. First, the small sample size limits the statistical power of the results, hence, further replication of these results in larger population is warranted. ADHD symptoms were assessed by a parent-rated questionnaire on a continuum, rather than analyzed dichotomously according to a formal diagnosis by a specialist. Therefore, the results of this study may differ in a clinical sample. SO_2_ gaseous exposure could have greater temporal variability, therefore, the results should be interpreted cautiously. In addition, DNAm measured in the peripheral blood may not reflect the methylation signature in the brain, as epigenetic markers are tissue specific. In the absence of gene expression data, direct conclusions about the transcriptional consequences of the DNAm changes could not be made. Despite these limitations, our study also has notable merits. We employed a prospective study design, with prenatal exposure assessment, methylation profiles at age 2 and 6, and longitudinal neurocognitive outcome measurements at ages 4, 6 and 8. Investigation of the effect of methylation provided insight into the mechanism underlying the association between air pollution and ADHD. The longitudinal study design enabled identification of susceptible window period of DNAm changes in association with prenatal environmental exposure, which was earlier childhood in this study. We adjusted for potential covariates related to both the exposure and outcome, including both maternal and childhood characteristics.

## Conclusion

In this study, we found that prenatal exposure to SO_2_ contributed to differential methylation at a CpG site located within *INS-IGF2* at age 2, which, in turn, was associated with ADHD symptoms at ages 4, 6 and 8 years. These findings indicate that an epigenetic mechanism involving methylation could underlie the relationship between the toxicity of SO_2_ and neurodevelopment in children.

## Methods

### Study population

Study participants were selected from the Environment and Development of Children (EDC) Study, a prospective cohort study that investigated the environmental effects on growth and neurodevelopment^59^. Mothers participating in the Congenital Anomaly Study cohort were contacted during 2012–2015 after birth, and a total of 726 mother–child pairs were recruited for EDC study, followed by regular follow-up at 2-year intervals (i.e. 2, 4, 6 and 8 years). We collected epidemiological information, anthropometric characteristics, and neurocognitive outcomes biennially. ADHD symptoms were evaluated at ages 4, 6, and 8. Among these children, 329 had information on ADHD symptoms all at ages 4, 6 and 8.

In a sub-cohort, we selected 60 children at age 2 and analyzed DNAm from whole blood at age 2 and 6 repeatedly. Similar to the main EDC cohort, we examined neurocognitive functions including symptoms of ADHD at ages 4, 6 and 8. Among 60 children, 54 at age 4, 60 at age 6, and 57 at age 8 were assessed for ADHD symptoms. Those assessed at these ages were included in the main analysis (n = 51).

### Ethical statements

The methods of this study were approved by the Institutional Review Board of Seoul National University College of Medicine (IRB No. 1201-010-392) and was conducted according to the guidelines and regulations of the Declaration of Helsinki. Informed consent was obtained from mothers according to the Institutional Review Board of Seoul National University College of Medicine (IRB No. 1201-010-392).

### Measurement of air pollution

We used the levels of air pollution including district-specific monthly SO_2_ as the main exposure. The levels of SO_2_ were collected from the publicly available data from Air Korea (http://www.airkorea.or.kr/eng) from the Korea Ministry of Environment, which monitors the hourly air pollution concentration levels at 257 stations nationwide. We assigned air pollution levels to the various pregnancy stages, such as the 1st, 2nd, 3rd trimester, as a proxy of exposure to pregnant women based on the residential address. The SO_2_ levels were measured from the nearest monitoring station from the residential address of each participant. The nearest monitoring stations was assigned according to the Euclidean distance between each residential address and the closest monitoring station using ArcGIS.

### DNAm analysis

#### Bisulfite sequencing and microarray

The Illumina Infinium HumanMethylation EPIC BeadChip (850 K) was used for the samples from the 2-year-olds, and the Illumina Infinium HumanMethylation 450 K BeadChip was used for the samples of the 6-year-olds (Illumina, San Diego, CA, USA). Detailed experimental procedures are presented in the Supplementary Materials.

#### Quality control and probe filtering

Array CpG probes which had a detection p-value > 0.05 in more than 25% of samples were filtered out. Filtered data were normalized using the Beta Mixture Quantile (BMIQ) method^60^ and corrected for batch effect using ComBat package in R. CpGs with at least one “not-available” (NA) values for the normalization were excluded, leaving 865,688 CpGs from the EPIC BeadChip (850 K) at age 2, and 460,960 CpGs from the 450 K BeadChip at age 6. Among these CpGs, we selected the overlapping 430,101 CpGs. After excluding SNP-associated CpGs (SNP distance ≤ 1 or SNP minor allele frequency ≥ 0.05), CpGs corresponded to non-CpG loci or the X or Y chromosomes, and CpGs at cross-reactive probes, and multimodal CpGs, 326,898 CpGs were finally selected for analysis (Fig. S1). We identified multimodal CpGs via the Dip test^61^ using diptest R package^62^ (Dip test’s p-value < 0.05).

### Systematic review of literature and selection of candidate CpGs

We selected candidate CpGs through a systematic literature review. The detailed procedure of systematic review is shown in the Supplementary Materials.

### SNP Genotyping

We used Axiom^®^2.0 Reagent Kit (Affymetrix Axiom^®^2.0 Assay User Guide) according to manufacturer’s protocol; the detailed procedure is provided in the Supplementary Materials. We used Korean Chip (K-CHIP) available from the K-CHIP consortium to produce Genotype data. K-CHIP was designed by the Center for Genome Science, Korea National Institute of Health, Korea (4845-301, 3000-3031).

### ADHD symptom screening

To evaluate ADHD symptoms in children at ages 4, 6, and 8, the Korean version of the ARS IV (K-ARS) was completed by the parents^63,64^. K-ARS is a standardized screening tool for ADHD symptoms in Korean children^65^ and has shown validity and reliability^66^. K-ARS, which has also been used in relation to environmental exposure^67^, is composed of 18 questions corresponding to the diagnostic criteria of ADHD according to the Diagnostic and Statistical Manual of Mental Disorders, 4th Edition. Nine questions evaluate inattention, and the other 9 are related to hyperactivity and impulsivity. Each item is rated from 0 to 3, with a total score ranging from 0 to 54^68^.

### Covariates

Covariates were pooled from the literature review^69–71^. Covariates included maternal age at pregnancy, maternal educational level (middle school graduate, high school graduate, college graduate, or graduate school attendance), maternal smoking status during pregnancy (current smoker, ex-smoker, never-smoker), ETS during pregnancy (yes/no), child’s sex, gestational age (weeks), multiple births (singleton or twin/triplet), season of child’s birth, cell type fraction, and maternal IQ evaluated by the short version of the Korean Wechsler Adult Intelligence Scale^72^. Cell type distribution, defined as the fraction of CD8 + T cells, CD4 + T cells, natural killer (NK) cells, B cells, monocytes, and neutrophils, was estimated by using the adult leukocyte reference panel^73^ and Minfi R package^74^.

### Statistical analysis

We investigated the association between trimester-specific prenatal exposure to SO_2_ (ppm) and ARS at ages 4, 6 and 8 in the cohort of 329 mother–child pairs by linear regression analysis by adjusting for maternal age at pregnancy, mother’s education levels, maternal smoking, maternal ETS, and season of child’s birth. Covariates were selected based on directed acyclic graph^75^ using the publicly available program (http://www.dagitty.net/) (Fig. S5).

In a sub-cohort (n = 51), we investigated the association between prenatal trimester-specific exposure to SO_2_ (ppm) and DNAm at the CpGs selected from the literature review (n = 375) at ages 2 and 6, using multivariable linear regression. We expressed the changes in DNAm per interquartile range (IQR) increase in SO_2_. The regression model for the association between prenatal SO_2_ and DNAm was adjusted for maternal age at pregnancy, mother’s educational level, maternal smoking, ETS during pregnancy, and season of the child’s birth (Fig. S6). To investigate the association between postnatal SO_2_ exposure and DNA methylation levels, we used a linear regression model for the average SO_2_ level during age 2 (12 months) and DNAm at age 2, and the average SO_2_ level during age 2, 4 and 6 each and DNAm at age 6, adjusted for mother’s age, postnatal ETS, postnatal mother’s smoking, and the season of birth. To evaluate the effects of cumulative SO_2_ exposure, we have analyzed the association between SO_2_ exposure at ages 2–4 years and 2–4–6 years and DNAm at age 6. SO_2_ exposure at ages 2–4 years was taken from the average of SO_2_ exposure at ages 2 and 4 years, and SO_2_ exposure at 2–4-6 years was taken from the average of SO_2_ exposures at age 2, 4 and 6 years. Benjamini–Hochberg method^76^ was used to correct the effects of multiple comparisons by using FDR. We also performed EWAS for 326,898 CpGs at ages 2 and 6 with prenatal SO_2_ exposure by trimester, adjusted by the same covariates.

Furthermore, we investigated the association between an increase in DNAm at prenatal SO_2_ exposure-associated CpGs at ages 2 and ARS at ages 4, 6, and 8, after adjusting for mother’s age at pregnancy, mother’s education level, maternal smoking, ETS during pregnancy, season of the child’s birth, maternal IQ, the child’s sex, preterm birth, and multiple births (Fig. S7).

WGCNA was used to detect co-methylated modules (clusters of CpGs) using WGCNA R package^77,78^. Using beta-value of methylation at the CpGs from candidate gene analysis, weighted co-methylation networks (module) were identified. For each module, hierarchical clustering was performed for all samples, and the dendrogram were grouped into modules. Then the correlation was tested between the modules and trimester-specific SO_2_ exposure during pregnancy, using Pearson correlation coefficient. We selected the module with a significant correlation with exposure variables, then pooled CpGs corresponding to the module of interest. Next, the methylation levels at these CpGs were used to detect co-methylation module, and the correlation between the modules and ADHD symptom scores at ages 4, 6 and 8 were then analyzed. The networks between CpGs were visualized using Cytoscape ver 3.8.2. (https://cytoscape.org/).

We investigated the mediation effect of DNAm at the significant CpGs on the association between prenatal SO_2_ and ARS in childhood using Mediation R package^79^. Indirect effect indicates the effect of prenatal exposure to SO_2_ affecting ARS through DNAm, whereas direct effect refers to the effect of prenatal SO_2_ exposure directly affecting ARS, not through DNAm. The total effect is defined as the sum of indirect and direct effects.

To identify mQTL, we searched for SNPs positioned within 100 kb window to either side of the significant CpGs, then analyzed the correlation between DNA methylation levels at the CpGs and SNP genotypes. The p-value for the correlation between genotype and DNAm levels was calculated using the Kruskal–Wallis test. Multiple comparisons were corrected via FDR.

For functional enrichment analysis, Gene Ontology (GO) terms and Reactome cell signaling pathways were identified using topGO R package^80^ and ReactomePA R package^81^.

## Supplementary Information

Supplementary Information.

Supplementary Tables.

## Data Availability

The data that support the findings of this study are available from Environment and Development of Children (EDC) cohort. The datasets used during the current study are available from the corresponding author on reasonable request.

## Abbreviations

ADHD: Attention deficit hyperactivity disorder
ARS: ADHD rating scale
DNAm: DNA methylation
ETS: Environmental tobacco smoking
EWAS: Epigenome-wide association study
FDR: False discovery rate
*GFI1*: Growth factor-independent 1 transcriptional repressor
GO: Gene ontology
IQR: Interquartile range
NK: Natural killer
