# Supplementary material for: DNA methylation is associated with prenatal exposure to sulfur dioxide and childhood attention-deficit hyperactivity disorder symptoms
Source: Sci Rep. 2023 Mar 1;13:3501. doi: 10.1038/s41598-023-29843-y (PMC9977725; doi:10.1038/s41598-023-29843-y)
Supplement: Supplementary file 1 — Supplementary Information. [file 41598_2023_29843_MOESM1_ESM.pdf]

Supplementary Materials

DNA methylation is associated with prenatal exposure to sulfur dioxide and childhood attention-deficit hyperactivity disorder symptoms

Yoon-Jung Choi[1,2,3#], Jinwoo Cho[4#], Yun-Chul Hong[2,3,5], Dong-wook Lee[2,3,6], Sungji Moon[2], Soo Jin Park[7], Kyung-shin Lee[2,3,8], Choong Ho Shin[9], Young Ah Lee[9], Bung-Nyun Kim[10], Zachary Kaminsky[11,12,13], Johanna Inhyang Kim[14*], Youn-Hee Lim[2,15*]

[1]National Cancer Center Graduate School of Cancer Science and Policy, Goyang, Republic of Korea

[2]Department of Preventive Medicine, Seoul National University College of Medicine, Seoul, Republic of Korea

[3]Environmental Health Center, Seoul National University College of Medicine, Seoul, Republic of Korea

[4]Department of Statistics, University of Pittsburgh, Pittsburgh, PA, USA

[5]Institute of Environmental Medicine, Seoul National University Medical Research Center, Seoul, Republic of Korea

[6]Public Healthcare Center, Seoul National University Hospital, Republic of Korea

[7]Department of Surgery, Wonkwang University Sanbon Hospital, Gunpo, Republic of Korea

[8] Public Health Research Institute, National Medical Center, Republic of Korea

[9]Department of Pediatrics, Seoul National University College of Medicine, Seoul National University Children's Hospital, Seoul, Republic of Korea

[10]Division of Children and Adolescent Psychiatry, Department of Psychiatry, Seoul National University Hospital, Seoul, Republic of Korea

[11]Institute of Mental Health Research, University of Ottawa, Ottawa, Canada

[12]Department of Cellular and Molecular Medicine, University of Ottawa, Ottawa, Canada

[13]Department of Psychiatry and Behavioral Sciences, Johns Hopkins University School of Medicine, Baltimore, USA

[14]Department of Psychiatry, Hanyang University Medical Center, Seoul, Republic of Korea

[15]Section of Environmental Epidemiology, Department of Public Health, University of Copenhagen, Copenhagen, Denmark

[#] These authors have contributed equally to this work and share the first authorship.
[*] These authors have contributed equally to this work and share the last authorship.

[*]**Correspondence**

Johanna Inhyang Kim,

Department of Psychiatry, Hanyang University Medical Center,

222-1 Wangsimni-ro, Seongdong-gu, Seoul, 04763, Republic of Korea

Telephone: +82 2 2290 9469,

E-mail: iambabyvox@hanmail.net

Youn-Hee Lim

Section of Environmental Epidemiology, Department of Public Health, University of Copenhagen,

Øster Farimagsgade 5, 1014 København K, Denmark

Telephone: +45 35 33 68 62

E-mail: younhee.lim@sund.ku.dk

Results

Selection of candidate genes

By using predetermined criteria, we identified 29 studies from PubMed, 61 studies from EMBASE, 60 studies from Web of Science, 54 studies from Scopus, and 5 studies from manual bibliographic search. After excluding 113 duplicated studies, 96 studies were remained. We further excluded irrelevant studies, studies with insufficient information, and studies that did not satisfy the selection criteria, finally leaving 22 studies (Supplementary Figure 1).

We pooled a total of 597 CpG sites from these studies, then excluded duplicated CpG sites (n=22), leaving 575 CpG sites. As we had information on 326,898 CpG sites from the study samples, 375 overlapping CpG sites were finally selected for further statistical analysis.

Methods

DNAm analysis

Bisulfite sequencing and microarray

Whole blood samples were repeatedly obtained from 51 children at ages 2 and 6. The quality of the DNA samples was assessed by the NanoDrop® ND-1000 UV-Vis Spectrophotometer (Thermo Fisher Scientific, Wilmington, DE, USA). Samples with intact genomic DNA (gDNA) were selected after electrophoresis on a 1% agarose gel, followed by dilution to 50 ng/µL according to Quanti-iT Picogreen quantification (Thermo Fisher Scientific, Wilmington, DE, USA). A minimum of 500 ng of gDNA was bisulfate-converted using Zymo EZ DNAm kit (Zymo Research, Irvine, CA, USA).

The amplified (×1000) bisulfite-converted DNA was used on each BeadChip, and was fragmented and hybridized by 50 mer capture probes at each CpG locus. Primers were extended using Two-Color Extension Master Mix (Illumina, San Diego, CA, USA), followed by staining, washing, and coating. The image intensities were extracted by Illumina GenomeStudio software, and the image was read by the Illumina BeadArray Reader. Microarrays were conducted by Macrogen (Seoul, Korea).

The level of DNAm at a single CpG locus was estimated as a beta value, which was calculated by the ratio of the methylated signal intensity divided by the sum of methylated and unmethylated signal intensities at the 5th carbon, ranging from 0 (no methylation) to 1 (fully methylated). Background signals from the negative control were subtracted from each signal.

Systematic review of literature and selection of candidate CpGs

The following keywords were used to search titles from Pubmed, EMBASE, Web of Science, and Scopus from May 2 to May 9, 2021: ("ADHD" or "attention deficit hyperactivity disorder" or "inattentive" or "attention" or "hyperactive" or "hyperactivity") and ("DNAm" or "epigenome-wide association study" or "EWAS" or "epigenetics" or "epigenetic" or "CpGs" or "CpG"). The selection criteria were peer-reviewed original articles written in English which studied the epigenetic profiles of ADHD in human case-control studies or cohort studies. Animal studies, review articles, studies regarding genetic effects only, and studies that did not provide specific DNAm positions and only indicated gene names were excluded (Peter et al.,

2016).

We pooled significant or suggestive CpGs associated with ADHD diagnosis or scales from the selected studies from the systematic review. If a study only provided probe position instead of the exact probe name, we selected CpGs closest to the probe position. In case of studies reporting significant or suggestive differentially methylated regions (DMRs) associated with ADHD, we pooled CpGs located within the reported DMRs.

SNP Genotyping

200ng of genomic DNA was amplified and randomly fragmented into 25 to 125 base pair (bp) sequence. Initial gDNA amplification was reacted in 40㎕ reaction volume, which contained 20㎕ volume of genomic DNA in concentration of 10ng/㎕, and 20㎕ of Denaturation Master Mix. The initial amplification was performed for 10 minutes at room temperature with 130㎕ of Axiom 2.0 Neutral Soln, 225㎕ Axiom 2.0 Amp Soln and 5㎕ Axiom 2.0 Amp Enzyme. The amplification reactions were performed for 23 hours ± 1 hour at 37℃ to produce 200-1,100 base pair fragments, which were further fragmented to segments of 25-50 bp. The fragments were end-labeled using biotinylated nucleotides, followed by hybridization. The bound targets were washed to remove non-specific background noise caused by random ligation events. Polymorphic nucleotides were queried through a multi-color ligation event on the array surface. After ligation, the arrays were stained and imaged on the GeneTitan MC Instrument (Affymetrix, Santa Clara, CA, USA), and the images were analyzed by using Genotyping Console™ Software (Affymetrix, Santa Clara, CA, USA).

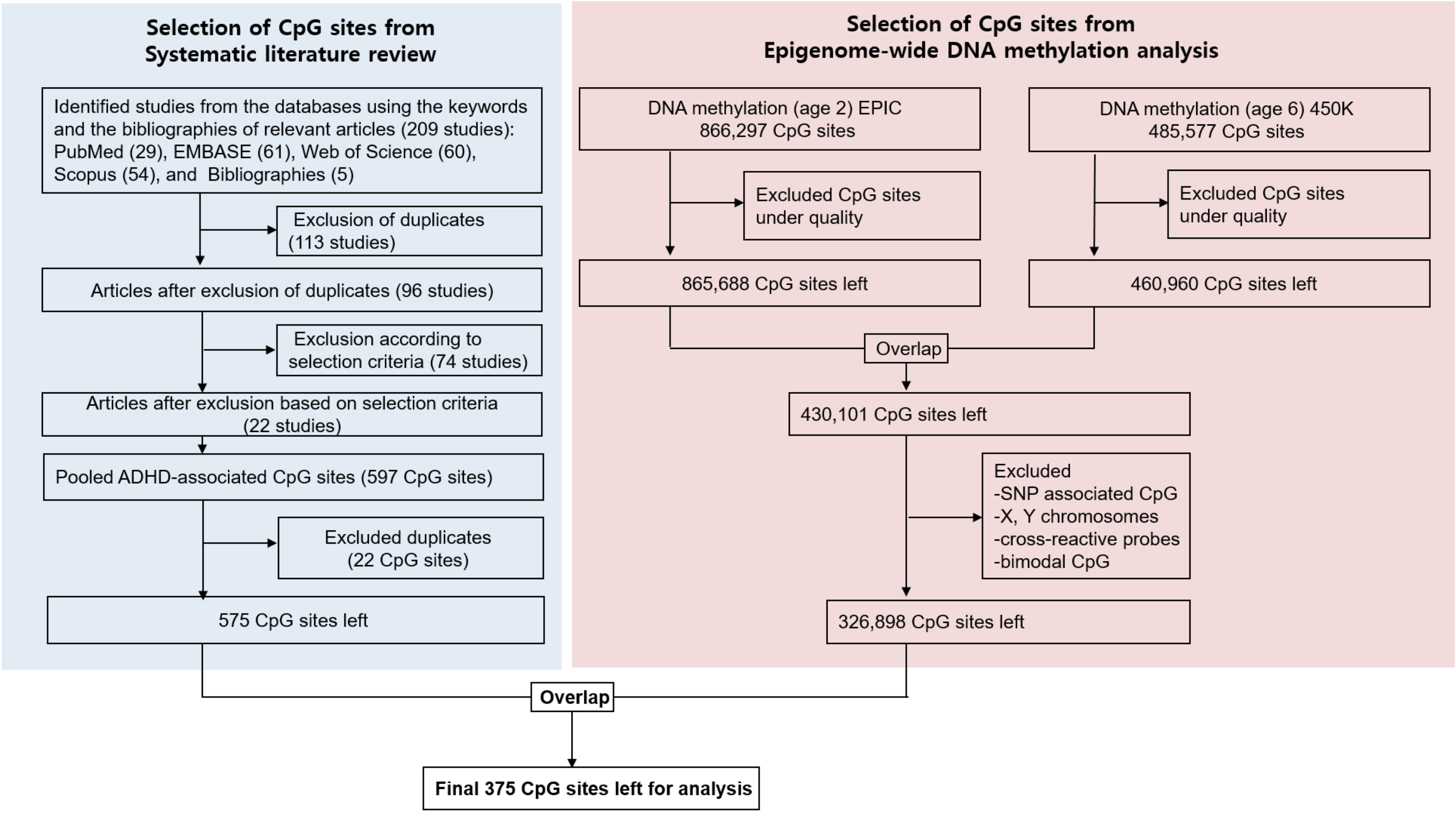

Figure S1. Flow diagram of selection of target CpG sites

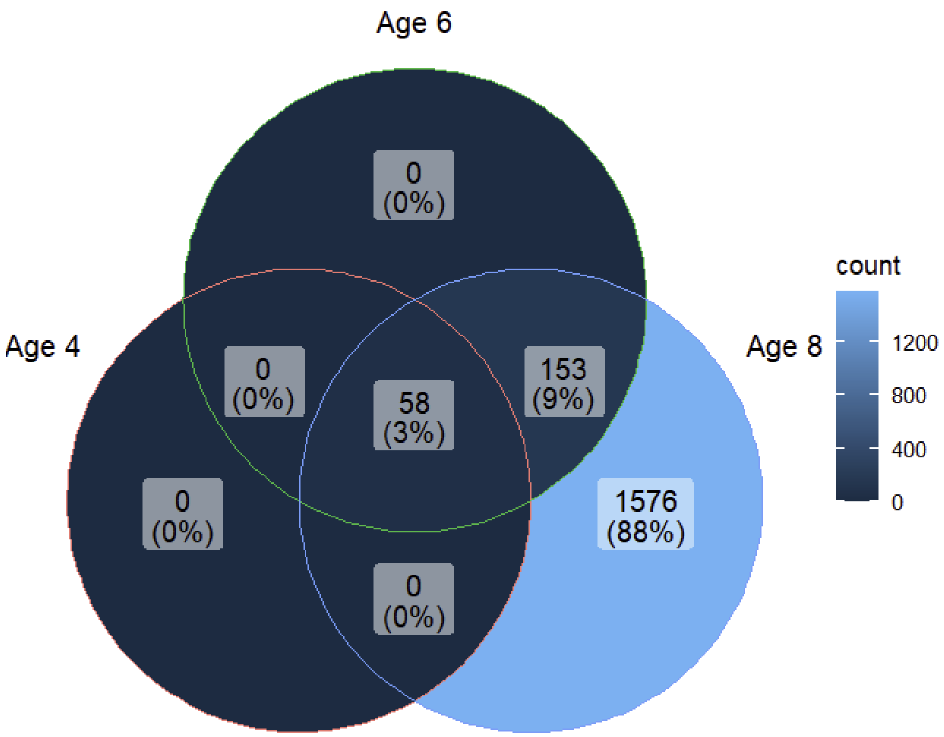

Figure S2. Venn diagram of CpG sites of DNA methylation at age 2 associated with ADHD rating scale at ages 4, 6, and 8 (58 CpGs).

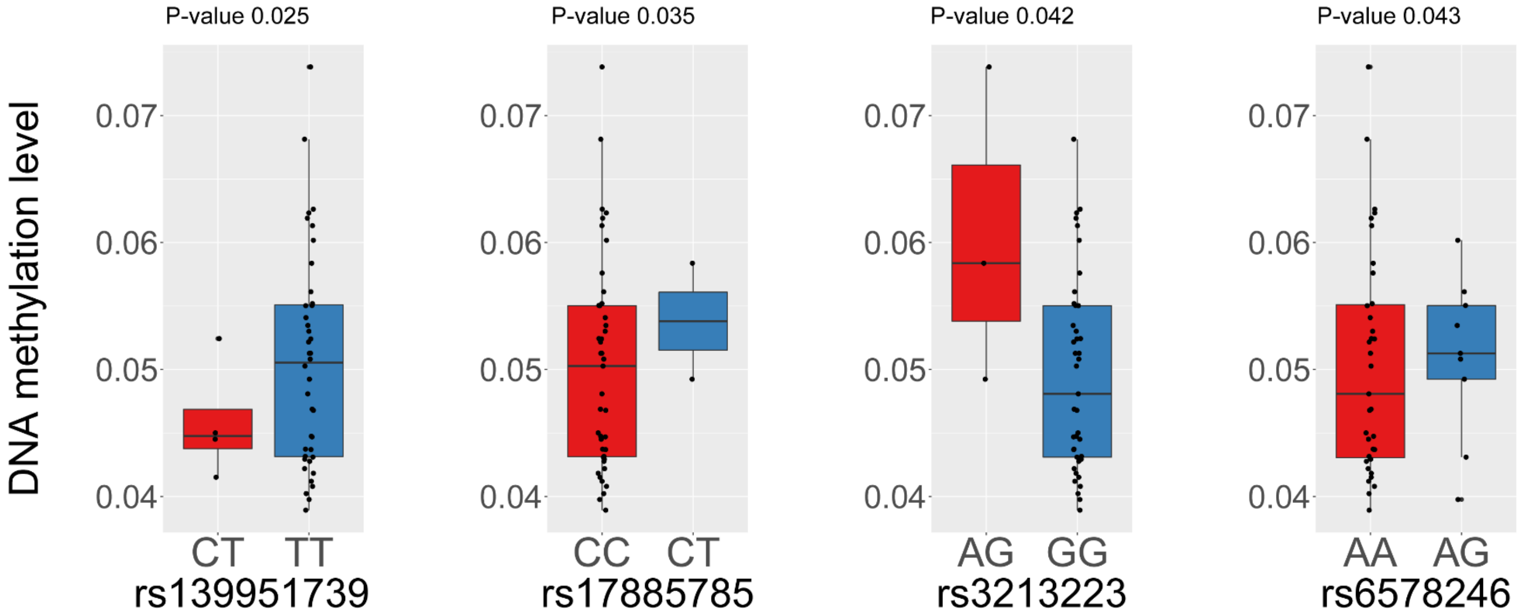

Figure S3. mQTL at cg07583420 (*INS-IGF2*) with 100kb window either side of the CpG site

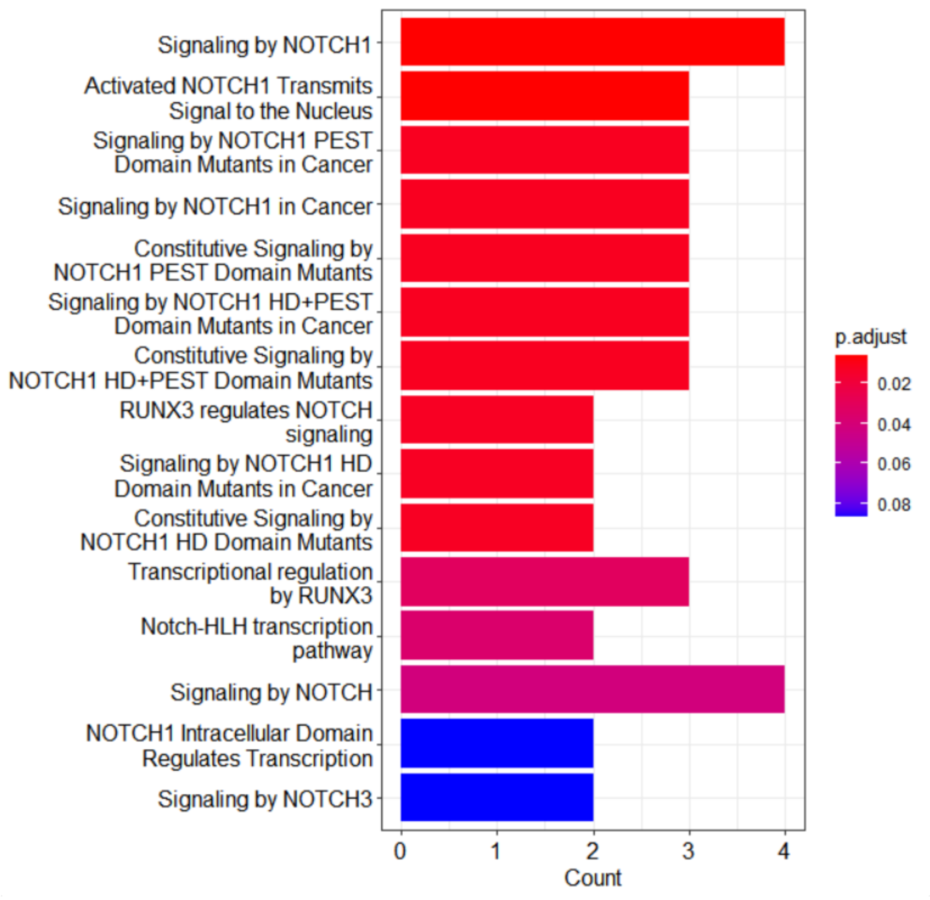

Figure S4. Reactome pathway analysis of genes annotated to CpG sites associated with prenatal SO_2 exposure during the 3rd trimester and ADHD rating scale at ages 4, 6, and 8 years

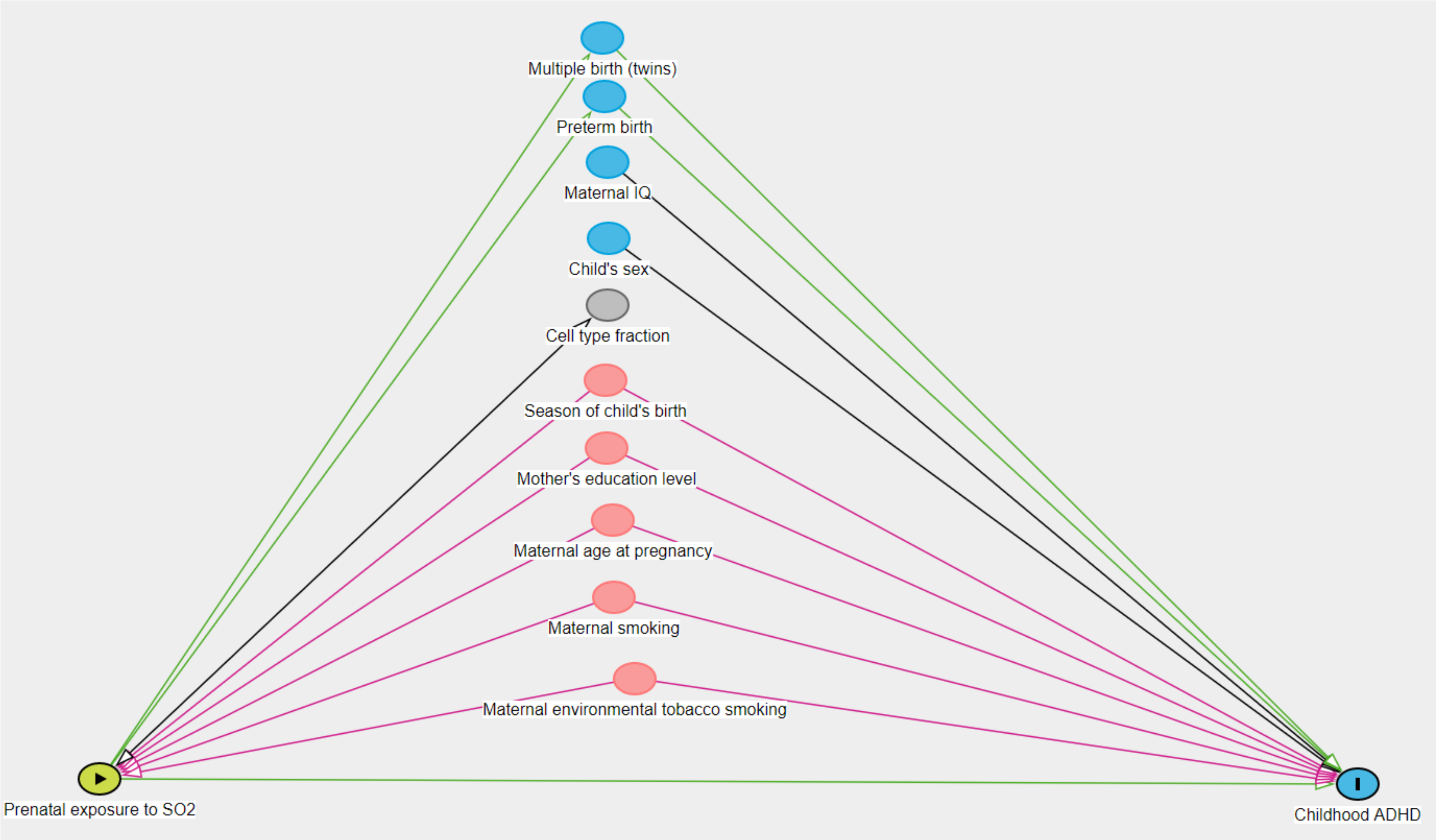

Figure S5. DAG for covariates used for the association between prenatal SO2 exposure and ADHD

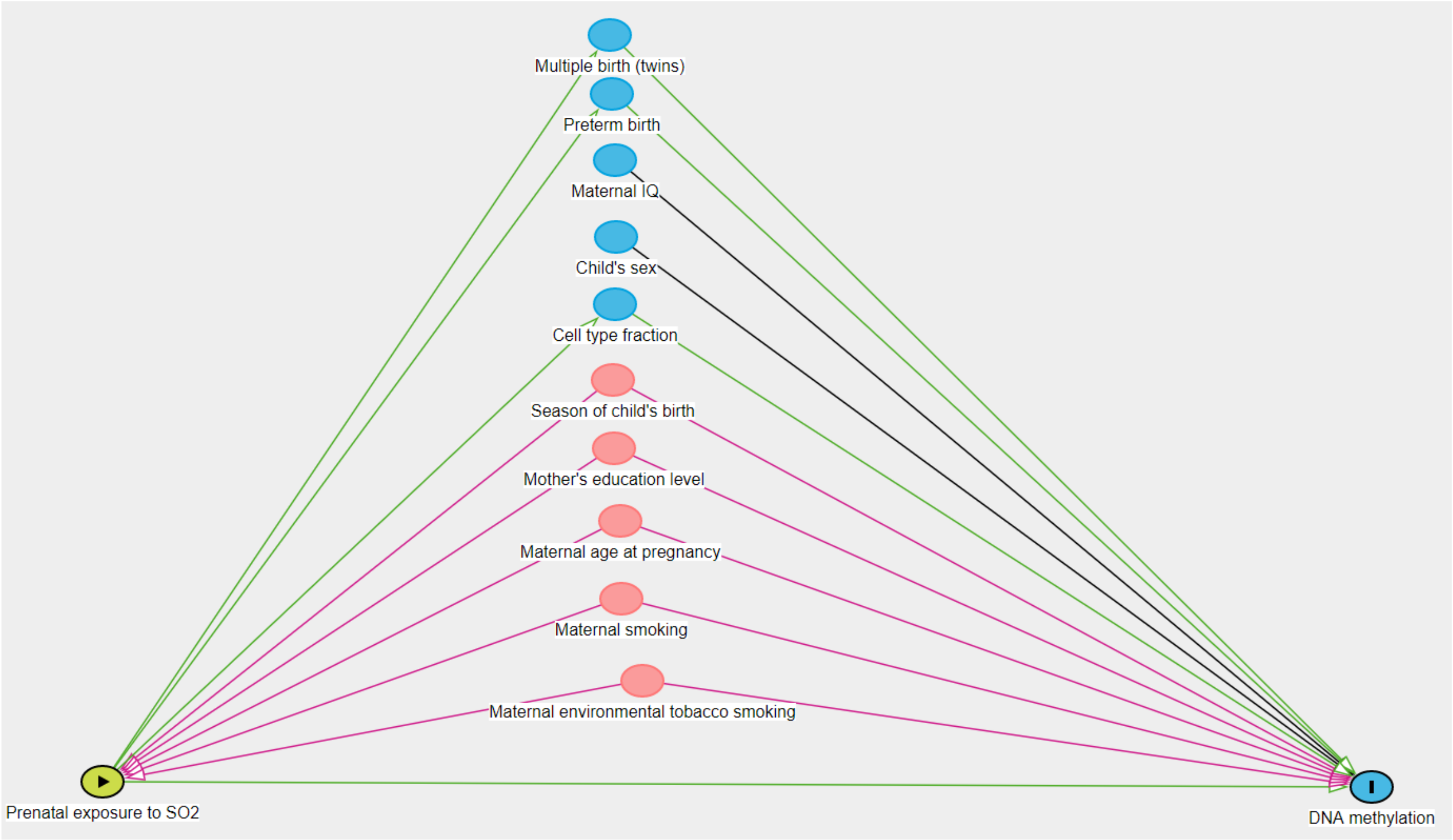

Figure S6. DAG for covariates used for the association between prenatal SO2 exposure and DNAm

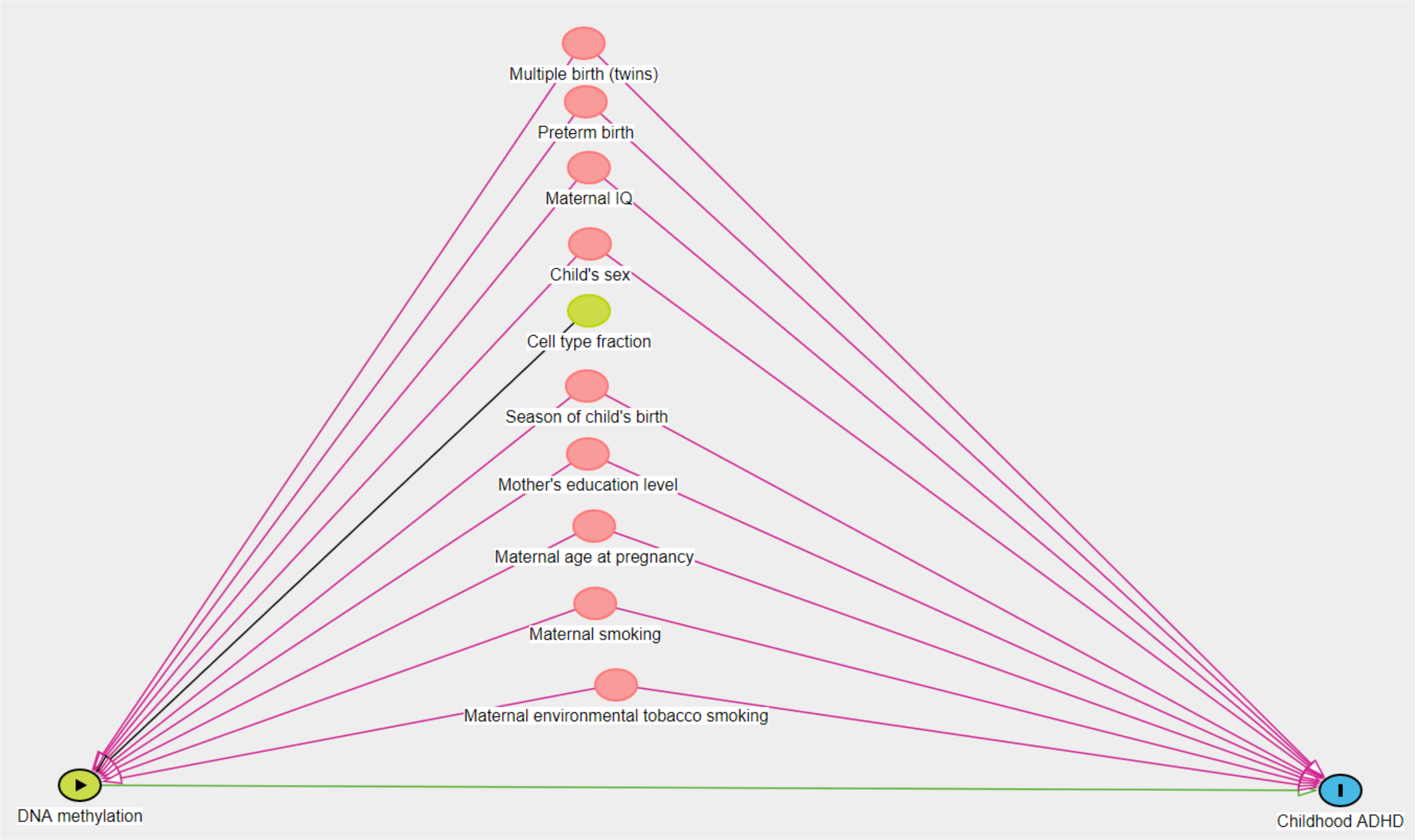

Figure S7. DAG for covariates used for the association between DNAm and ADHD
